# HPV Vaccination Champions: Evaluating a Technology-Mediated Intervention for Parents

**DOI:** 10.3389/fdgth.2021.636161

**Published:** 2021-02-15

**Authors:** Beth Sundstrom, Kathleen B. Cartmell, Ashley A. White, Nicole Russo, Henry Well, Jennifer Young Pierce, Heather M. Brandt, James R. Roberts, Marvella E. Ford

**Affiliations:** ^1^Department of Communication, College of Charleston, Charleston, SC, United States; ^2^Department of Public Health Sciences, Clemson University, Clemson, SC, United States; ^3^Department of Public Health Sciences, Medical University of South Carolina, Charleston, SC, United States; ^4^Department of Communication, College of Charleston, Charleston, SC, United States; ^5^South Carolina Cancer Alliance, Columbia, SC, United States; ^6^Mitchell Cancer Institute, University of South Alabama, Mobile, AL, United States; ^7^St. Jude Children's Research Hospital and Comprehensive Cancer Center, Memphis, TN, United States; ^8^Department of Pediatrics, Medical University of South Carolina, Charleston, SC, United States; ^9^Hollings Cancer Center, Medical University of South Carolina, Charleston, SC, United States

**Keywords:** human papillomavirus, technology-mediated intervention, champions, parents, social media

## Abstract

Human papillomavirus (HPV) vaccination prevents 6 HPV-related cancers in men and women. Yet, rates of HPV vaccination among adolescents in the United States lag behind other developed nations, revealing a significant public health issue. This feasibility study tested a collaborative online learning environment to cultivate HPV vaccination champions. A 3-month training program recruited parents to serve as proponents and social media influencers to identify solutions to overcome barriers to HPV vaccination. A mixed methods study design included a pretest survey, three online asynchronous focus groups, a posttest survey, as well as a longitudinal follow-up survey at 6 months. Participants included 22 parents who self-identified as female (95.4%) and white (90.9%). Overall, there was a statistically significant difference in knowledge of HPV and HPV vaccination between pretest and posttest (*p* = 0.0042). This technology-mediated intervention increased parents' confidence and motivated them to speak more freely about HPV vaccination in-person and online with others in their social networks. Participants identified prevalent misinformation about HPV vaccination and learned how to effectively craft messages to address concerns related to safety and side effects, gender, understanding of risk, and sexual activity. Objective measures and qualitative open-ended assessment showed high intervention engagement and treatment satisfaction. All participants (100%) indicated that they enjoyed participating in the intervention. The effectiveness of this feasibility study suggests that social media is an appropriate platform to empower parents to counter vaccine hesitancy and misinformation through HPV vaccination information that is simple and shareable in-person and online.

## Introduction

The human papillomavirus (HPV) is the most prevalent sexually transmitted infection (STI) in the United States, with 79 million Americans currently infected with the virus (1). The majority of sexually active men and women in the U.S. will be infected with HPV during their lifetime, and 14 million Americans become infected each year (1). While most HPV infections will not cause symptoms or result in health problems, persistent infections can cause genital warts and six types of cancer. HPV infection is linked to six different types of cancer and is estimated to cause more than 90% of cervical and anal cancers; 70% of vaginal, vulvar, and oropharyngeal cancers; and 60% of penile cancers. Every year, HPV is estimated to cause ~35,900 of the 45,300 new cases of HPV-associated cancer found in women and men (2). In South Carolina, more than 580 new cases of HPV-related cancers are diagnosed each year (3).

The HPV vaccine is critical to reduce HPV infection rates and HPV-related cancers. A vaccine to prevent HPV has been available in the U.S. since 2006. Gardasil®9 (Merck, Inc) has been offered in the U.S. since 2016 and is currently the only HPV vaccine available in the U.S. The Centers for Disease Control and Prevention (CDC) recommend that all children ages 11 and 12 receive two doses of HPV vaccine. Adolescents who receive the first dose of the HPV vaccine at age 15 or older or who are immunocompromised require three doses. The HPV vaccine is recommended for all men and women up to age 26 and is approved for some people up to the age of 45 (4). It provides protection from nine HPV types that cause genital warts and cervical, vaginal, vulvar, anal, oropharyngeal, and penile cancers. The HPV vaccine is safe and effective with only minor side effects, such as pain or swelling at injection site, fever, headache, nausea, and fainting (5). More than 100 million doses of HPV vaccine have been distributed in the U.S. and continuous monitoring further strengthens the evidence of the vaccine's safety and effectiveness (5).

Research shows that social media play a role in spreading the global anti-vaccination movement (6). Parents are exposed to negative messages about vaccination on social media (7). In South Carolina, a recent content analysis of social media found that online messages perpetuated barriers to HPV vaccination, including fears about vaccine safety and concerns about harmful side effects (8). A study of parents who sought vaccine information on the internet found that they were more likely to have lower perceptions of vaccine safety, vaccine effectiveness, and disease susceptibility compared with parents who did not seek vaccine information on the internet (9). Exposure to negative opinions about HPV vaccines on social media led to increased anti-vaccination posts, whereas neutral or positive information did not have the same impact on users' posts (10). In fact, mothers who are against childhood vaccinations are more likely to engage in communication about the issue, while those who support vaccinations remain silent (11). Researchers argue that social media platforms offer an important venue for sharing science-based information about the safety of vaccines and suggest that social media users may be able to debunk myths and inactivate misinformation (12).

HPV vaccination interventions have primarily focused on adolescents, parents, and clinicians. In addition to their robust use of social media, women tend to be the health decision makers in their families. Research shows that mothers serve as the primary decision makers for adolescents receiving HPV vaccination (13–15). Parents' social networks influence their vaccination decision-making by offering information and advice (16). Past HPV vaccine interventions targeted at parents have been effective in increasing knowledge and acceptance of the HPV vaccine, as well as intention to vaccinate children (17, 18). Shoup et al. created an effective social media intervention tool to address parental concerns about vaccination and improve childhood immunization rates (19). Another social media intervention successfully improved childhood vaccine acceptance among pregnant women (20).

Although Americans continue to report high levels of trust in health care providers and government health agencies, a recent study found that the social media accounts of patients and support groups were more influential than physician, academic society, and clinic accounts (21). Health education interventions have utilized social media champions to successfully promote health messages while other health interventions have demonstrated the success of preparing parents to be advocates in their own communities by providing them with information that can be used in discussions with other parents to improve vaccination uptake (22, 23). Research shows that cultivating champions is an effective implementation strategy to promote uptake of an evidence-based intervention (24–27).

Building on the evidence that social media can be a powerful platform for promoting vaccination, the current study was conducted as part of a statewide initiative to raise HPV vaccination rates in South Carolina. This research answers the call to action by researchers to assist parents who support vaccination to speak out easily and often by providing information that is simple and shareable online (11). According to Dr. Aaron E. Carroll, professor of pediatrics and associate dean at the Indiana University School of Medicine, “It seems important to engage the public more, and earn their trust through continued, more personal interaction, using different platforms and technologies. Dropping knowledge from on high-which is still the modus operandi for most scientists—doesn't work” (28). The purpose of cultivating HPV vaccination champions is to develop a collaborative online learning environment to increase HPV vaccination by training and supporting parents to serve as proponents and social media champions in order to overcome barriers to HPV vaccination.

## Materials and Methods

### Design

A mixed methods study examined the feasibility of a technology-based intervention among parents in South Carolina. This study included the implementation and evaluation of a 3-month online training designed to cultivate HPV vaccination champions. The intervention was adapted from a successful theory-based, technology-mediated HPV vaccination awareness intervention for college students (29). Recruitment methods are described below. Participants joined a private Facebook group, received bi-weekly emails with facts about HPV vaccination, and attended two online webinars about HPV vaccination. These communication strategies mirrored Shoup et al.'s successful social media intervention that facilitated interaction with parents through a newsletter, blog, discussion forum, chat room, and portal to ask questions (19). In the current study, eight bi-weekly emails were distributed through MailChimp (Rocket Science Group, LLC, Atlanta, GA), which tracked newsletter open rate. The research team posted to the private Facebook message board ~5 times each week. Researchers posted information and facts about HPV vaccination, shared current news stories, and promoted engagement through polls and discussion prompts. Participants completed a pretest survey, three online asynchronous focus groups, a posttest survey, as well as a longitudinal follow-up survey at 6 months.

### Participants and Setting

Participants included parents living in South Carolina who were committed to increasing HPV vaccination and were *active users* (post at least once per week, log-in at least once per day) of Facebook **and** Twitter. A screening tool was used prior to enrollment in the study. For this feasibility study, we recruited participants who were committed to increasing HPV vaccination and dedicated to starting conversations (online and in-person) and answering questions about HPV vaccination in their social networks. Participants were recruited through word of mouth, email messages, social media posts, and at relevant meetings and events. As a result, snowball sampling occurred when participants recommended additional participants. Participants received an incentive for their time and effort in the study, including $100 after completing the 3-month training and $20 for completing the longitudinal follow-up at 6 months. Informed consent was obtained through Qualtrics by all parents prior to participation.

### HPV Vaccination Champions Intervention

**Figure 1:**
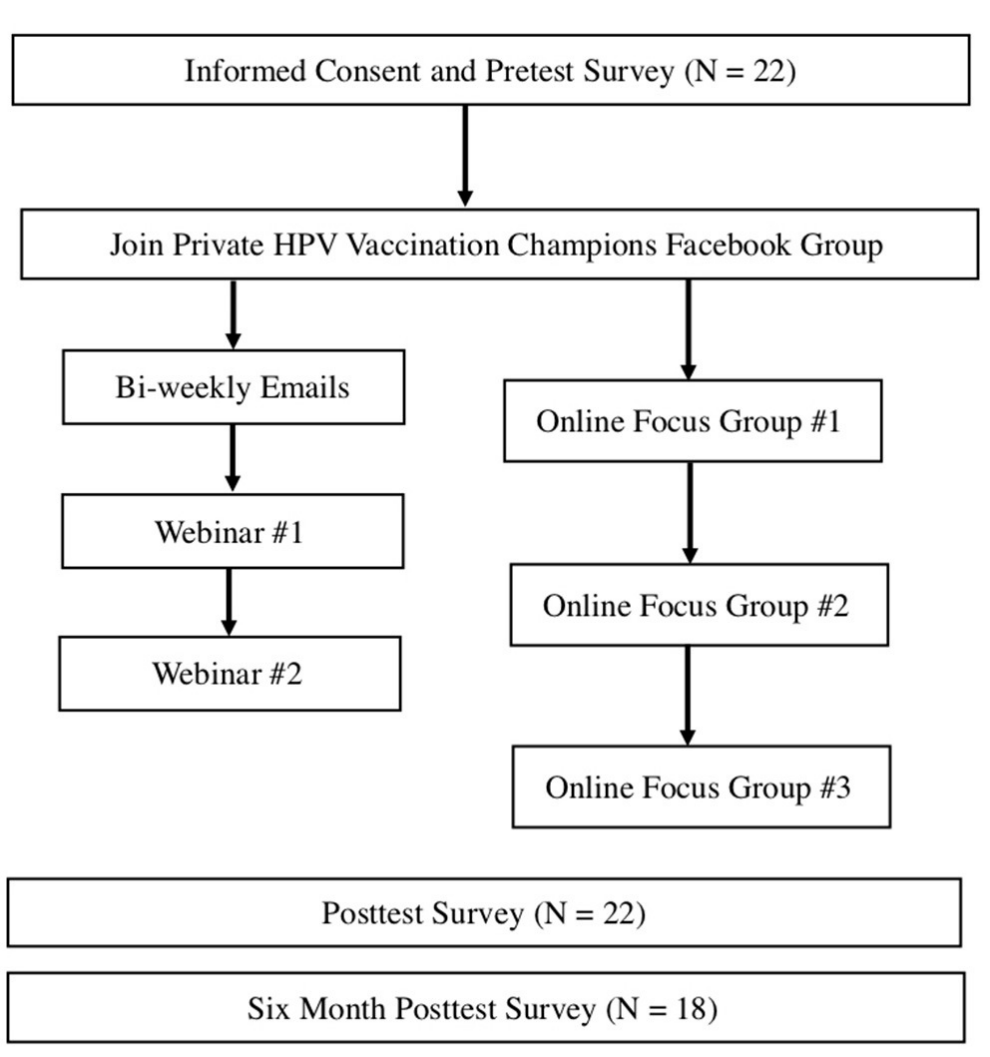
HPV vaccination champions intervention.

The development of the intervention was informed by best practices in implementation science (26, 30) and based on a successful technology-mediated HPV vaccination awareness intervention for college students (29). Messages and health education information were adapted for parents based on formative audience research (8, 31, 32). Content was delivered through a private Facebook group, bi-weekly emails with facts about HPV vaccination, and two online webinars about HPV vaccination (see Figure 1). Eight emails were sent to participants on a bi-weekly basis that included topics such as: What is HPV? Who is at risk? Can HPV and HPV-related cancers be prevented? (see Table 1). Participants attended two live online webinars lasting ~1 h each, which were also archived on Facebook. The first webinar covered “What is HPV Vaccination?” and addressed common misconceptions. The second webinar covered “How to be an Effective Spokesperson for HPV vaccination” online and in-person. Participants engaged in a private Facebook group, responding to polls, posting messages, and asking questions of one another (peer-to-peer), as well as experts on the research team.

**Table 1:** Bi-Weekly emails with facts about HPV vaccination (*n* = 22).

**Email**	**Topics**	**Open**
1	What is HPV?	15 (68.2%)
2	Who is at risk for HPV?	7 (31.8%)
3	Can HPV and HPV-related cancers be prevented?	10 (45.5%)
4	Does HPV cause symptoms?	9 (40.9%)
5	What are the risks and benefits of HPV vaccination?	8 (36.4%)
6	Who should get the HPV vaccine?	12 (54.5%)
7	Where is the HPV vaccine available?	8 (36.4%)
8	How can I get involved in HPV vaccination efforts in our state?	14 (63.6%)

The goals of the 3-month online training were to help HPV vaccination champions explain HPV vaccination recommendations, discuss the importance of HPV vaccination and the risks of HPV-related cancers and disease, describe ways an ambassador can increase HPV vaccination by supporting vaccination and overcoming barriers, provide examples of activities to engage in as an ambassador (e.g., letters to the editor/op-eds; social media posts), and understand resources to support champions. The webinars were designed to respond in real time to the questions and concerns participants expressed during online focus groups and the private Facebook group.

### Questionnaires

Participants completed a questionnaire at baseline (pretest) and post-intervention at 3 months (posttest) and 6 months (longitudinal follow-up). The questionnaires investigate awareness and knowledge of HPV and the HPV vaccine, attitudes and beliefs about HPV and the HPV vaccine, and behavior/behavioral intention regarding HPV and the HPV vaccine. The measures were drawn from a variety of sources and have proven to be reliable and valid, including the Health Information National Trends Survey (HINTS), the Behavioral Risk Factor Surveillance System (BRFSS), TTM, HBM, and surveys of young adult populations (33–38), which enable comparison of our results from those of prior studies. Baseline demographic characteristics self-reported at pretest included age, gender, race, level of education, health insurance status, income, county of residence, and technology use. Surveys were administered online through Qualtrics (Provo, UT).

### Intervention Engagement

Engagement was assessed objectively on web-based platforms (Facebook and MailChimp). Engagement with bi-weekly emails was defined as the number of participants who opened the email, which was obtained from MailChimp metrics. Engagement with the private Facebook group was measured by the number of interactions, including post likes, comments, and original posts.

### Treatment Satisfaction

The online focus groups and the posttest and longitudinal follow-up questionnaires assessed how satisfied participants were with the training by rating its overall usefulness and likelihood of recommending it to a friend. Level of satisfaction with specific intervention components (e.g., emails, Facebook group, and webinars) was also reported. All items were rated on an agreement-oriented 7-point Likert scale anchored with strongly agree and strongly disagree.

### Statistical Analysis

All data were reported as frequencies and response rates were reported as percentages of the total sample population. Basic descriptive statistics were used to describe the sociodemographic characteristics, participants' use of technology and participant retention rates. For analysis of technology use, cell phone included responses of “receive a text message on a cell phone,” “send a text message on a cellphone” and “use a cellphone to make or receive a voice call.” Hourly included responses of “about once an hour” and “more than once an hour.” Daily included responses of “about once a day” and “several times a day.” Weekly included responses of “up to about once a week” and “a few times a week.” Friedman's test was used to compare pretest, posttest and 6-month follow-up responses within participants' intention to vaccinate their child against HPV. Responses were ranked in order of 1= “I am unsure about my intention to get my child vaccinated” to 11= “My child has received all three shots of the HPV vaccine.” Exact McNemar's tests were used to compare pretest and posttest responses for each question of HPV and HPV vaccination knowledge. Responses were recoded as binary to designate correct answers as “1” and incorrect answers or “don't know” as zero. The overall average of correct responses was analyzed from composite scores calculated from the sum of correct responses per individual. A paired *T*-test was used to compare the pretest and posttest composite score for the overall scale, given the continuous distribution of these data. Wilcoxon Signed-Rank tests were used to compare pretest and posttest responses of HPV and HPV vaccination attitudes. Responses were ranked in order of 1 = strongly disagree, 2 = disagree, 3 = neutral, 4 = agree, 5 = strongly agree, and 6 = don't know. For analysis, agree included responses of “strongly agree” and “agree.” Disagree included responses of “strongly disagree” and “disagree.” For analysis of HPV vaccination influence, influence included responses of “strongly influenced” and “influenced.” For analysis of participant intervention experience, extremely included Likert scale responses of “4” and “5.” A *p-*value ≤ 0.05 was used to determine statistical significance for all analyses. All data analyses were conducted using SAS statistical software version 9.4 (SAS Institute, Cary, North Carolina).

### Focus Groups

Participants were invited to participate in three online asynchronous focus groups. Focus groups allow researchers to better understand socially constructed understandings of HPV and HPV vaccination. The first focus group addressed knowledge, attitudes, and beliefs about HPV vaccination. Questions included myths/misunderstandings about HPV vaccination. The second focus group addressed communicating about HPV vaccination, including challenges and opportunities to improve in-person conversations and constructing media messages. The third focus group asked specifically about the HPV vaccination champions intervention, including visibility of messages in the community, perceptions of messages, needs/preferences for future messaging, successes and opportunities for improvement.

Research shows that online focus groups include many advantages, such as convenience, accuracy of data, low costs, expanded geographic range, and increasing access to specific types of participants (e.g., parents), while preserving the quantity and quality of data collected during in-person groups (39, 40). In line with Levine et al.'s evidence-based approach, during a 5-day period, researchers posted one question per day on the private Facebook group and participants responded within a set time frame at their own pace (39). Since online focus groups require a skilled moderator, the first author moderated all focus groups and relied on response elicitation techniques, such as sharing summaries, offering feedback, and frequently encouraging comments to promote participation and engagement (40). Participants responded to questions at their convenience in the comfort of their homes, which can result in longer and more detailed responses and optimal group discussion than traditional focus groups (39, 40).

### Qualitative Analysis

Qualitative data analysis of the online focus groups was conducted using a constant comparative method (41). Researchers with graduate level qualitative training coded line-by-line, which allowed new concepts to emerge. A codebook was developed based on extant literature and emergent concepts. Axial coding identified cross-cutting themes and concepts in the data. Researchers met frequently throughout the implementation and evaluation of the intervention and reached unanimous consensus on conclusions emerging from the data.

This study was approved by the Institutional Review Board at the College of Charleston.

## Results

Participants included 22 parents with a median age of 40.2 ± 6.6 years. Most participants self-identified as female (95.4%) and white (90.9%). Participants lived in counties across South Carolina with representation from each of four regions in the state, including the Upstate Region, Midlands Regions, Lowcountry Region, and Pee Dee Region. All participants reported private health insurance coverage. Participants' education ranged from a high school diploma (4.5%), some college (9.1%), an undergraduate degree (27.3%), some graduate education (13.6%), and a graduate degree (45.4%). All participants reported an annual household income above $30,000 with the majority reporting $70,000 or more (63.6%). Among participants, 62.6% reported using a cell phone every day and 30.3% reported using it every hour. Almost half of participants (45.5%) reported using a computer every day with 9.1% reporting hourly use. The majority of participants reported using multiple devices every day (59.1%) with 22.7% reporting hourly use of multiple devices. All participants completed the posttest survey and the retention rate at the 6-month longitudinal follow-up was high (82%) (see Table 2).

**Table 2:** Baseline characteristics and retention rates.

**Characteristic**	**Total (*N* = 22)**
Age (*SD*), years	40.2 (6.6)
**Sex**, ***n*** **(%)**	
Female	21 (95.4%)
Male	1 (4.5%)
**Hispanic**, ***n*** **(%)**	
No	22 (100)
**Race**, ***n*** **(%)**	
White	20 (90.9%)
Black or African-American	2 (9.1%)
**South Carolina County**, ***n*** **(%)**	
Anderson	1 (4.5%)
Berkeley	2 (9.1%)
Charleston	7 (31.8%)
Chesterfield	2 (9.1%)
Darlington	1 (4.5%)
Dorchester	1 (4.5%)
Greenville	3 (13.6%)
Kershaw	1 (4.5%)
Oconee	1 (4.5%)
Sumter	3 (13.6%)
**Health Insurance Status**, ***n*** **(%)**	
Private Insurance	22 (100%)
**Education**, ***n*** **(%)**	
High school diploma/GED	1 (4.5%)
Some college education	2 (9.1%)
Undergraduate education	6 (27.3%)
Some graduate education	3 (13.6%)
Graduate degree	10 (45.4%)
**Household Income**, ***n*** **(%)**	
$30,000–$49,999	3 (13.6%)
$50,000–$69,999	5 (22.7%)
$70,000 or more	14 (63.6%)
**Technology Use (%)**	
Cell Phone^a^	
Hourly Use	30.3%
Daily Use	62.6%
Weekly Use	7.1%
Monthly Use	0%
Never Use	0%
Computer	
Hourly Use	9.1%
Daily Use	45.5%
Weekly Use	22.7%
Monthly Use	9.1%
Never Use	13.6%
Multiple Devices^b^	
Hourly Use	22.7%
Daily Use	59.1%
Weekly Use	13.6%
Monthly Use	4.5%
Never Use	0%
**Retention rates**, ***n*** **(%)**	
Posttest	22 (100%)
6-month follow-up	18 (82%)

a *Cell phone use includes responses of “receive a text message on a cell phone,” “send a text message on a cellphone,” and “use a cellphone to make or receive a voice call.”*

b *Hourly includes responses of “about once an hour” and “more than once an hour.”*

*Daily includes responses of “about once a day” and “several times a day.”*

*Weekly includes responses of “up to about once a week” and “a few times a week.”*

### HPV Vaccination Knowledge, Attitudes, and Behaviors

At baseline, half of participants (*n* = 11; 50%) reported “my child has received all shots of the HPV vaccine series,” 9 participants (40.9%) indicated “I plan to get my child vaccinated at the recommended age,” and 2 participants (9.1%) reported “I am unsure about my intention to vaccinate/I do not plan to get my child vaccinated in the next 6 months” (see Table 3). Following the intervention, two participants changed from being unsure to planning to vaccinate at the recommended age. There were no statistically significant differences between pretest, posttest and 6-month follow-up responses within participants (Table 3).

**Table 3:** Intention to vaccinate child against human papillomavirus (HPV).

	**Pre** **(*n* = 22) %**	**Post** **(*n* = 22) %**	**6-month follow-up** **(*n* = 18) %**
**Action Stage:**			
My child has received all shots of the HPV vaccine series	50%	50%	50%
**Preparation Stage:**			
I plan to get my child vaccinated at the recommended age	40.9%	50%	50%
**Contemplation Stage:**			
I am unsure about my intention to vaccinate/I do not plan to get my child vaccinated in the next 6 months	9.1%	0%	0%

^*^Friedman's test compared pre-post and 6-month follow-up responses for intent to vaccinate their child against HPV.

At pretest, all participants (100%) knew that HPV can be spread through sexual intercourse, HPV can cause an abnormal Pap (cervical cancer screening) test, and some types of HPV can cause cervical cancer. Fewer participants were aware that HPV can be spread through contact other than sexual intercourse (77.3%) and that some types of HPV can cause oral cancer (81.8%). Participants reported changes in knowledge from pretest to posttest, particularly learning that “some types of HPV can cause anal cancer,” “condom use does not fully protect against the spread of HPV” and “an HPV infection cannot be cured.” Overall, there was a statistically significant difference in the average of correct answers from pretest and posttest (*p* = 0.0042) (Table 4).

**Table 4:** Human papillomavirus virus (HPV) and HPV vaccination knowledge.

	**Pre** **(*****n*** **=** **22)**		**Post** **(*****n*** **=** **22)**		**Difference in change at posttest** (*p*-value)^*^
	***n***	**%**	***n***	**%**	
Some types of HPV can cause anal cancer.	17	77.3%	21	95.4%	4 (0.001)
Correct answer: **True**					
Condom use fully protects against the spread of HPV.	15	68.2%	19	86.4%	4 (0.0075)
Correct answer: **False**					
An HPV infection can be cured.	15	68.2%	19	86.4%	4 (0.0075)
Correct answer: **False**					
Some types of HPV can cause oral cancer.	18	81.8%	21	95.4%	3 (<0.001)
Correct answer: **True**					
HPV can be spread through contact other than sexual intercourse.	17	77.3%	20	90.9%	3 (0.0007)
Correct answer: **True**					
Some types of HPV can cause genital warts.	20	90.9%	22	100%	2 (<0.001)
Correct answer: **True**					
People who have been infected with HPV might not have symptoms.	21	94.4%	22	100%	1 (<0.001)
Correct answer: **True**					
HPV can cause an abnormal Pap (cervical cancer screening) test.	22	100%	22	100%	0
Correct answer: **True**					
HPV can be spread through sexual intercourse.	22	100%	22	100%	0
Correct answer: **True**					
Some types of HPV can cause cervical cancer.	22	100%	22	100%	0
Correct answer: **True**					
Women who get the vaccine still need regular Pap (cervical cancer screening) tests.	21	95.4%	21	95.4%	0
Correct answer: **True**					
Overall average of correct responses	9.54	10.50	0.0042		

* *Exact McNemar's tests compared pre-post responses for individual items, dichotomized as correct vs. incorrect/don't think. A paired T-test compared pre-posttest score for the overall scale.*

Participants reported high perceptions of HPV vaccination benefits, barriers and severity; however, they reported low susceptibility. Parents' attitudes about HPV and HPV vaccination mirrored the constructs of the Health Belief Model except related to perceptions of susceptibility. There were no statistically significant differences between responses (Table 5).

**Table 5:** Human papillomavirus (HPV) and HPV vaccination attitudes.

**Health belief model constructs**	**Pre** **(*n* = 22)** **%**	**Post** **(*n* = 22)** **%**
**Benefits:**		
“Getting the HPV vaccine would help my child stay healthy.”		
**Agree**	100%	100%
“Getting the HPV vaccine would benefit a significant other or partner.”		
**Agree**	100%	100%
“Getting the HPV vaccine would be a benefit to society.”		
**Agree**	100%	100%
**Severity:**		
“A vaccine that prevents a sexually transmitted infection is a good idea.”		
**Agree**	100%	100%
“A vaccine that prevents HPV-related cancer is a good idea.”		
**Agree**	100%	100%
“A vaccine that prevents genital warts is a good idea.”		
**Agree**	100%	100%
“Having genital HPV would make it difficult for someone to get a long-term sex partner.”		
**Disagree**	59.1%	54.5%
**Barriers:**		
“My healthcare providers would approve of my child getting the HPV vaccine.”		
**Agree**	100%	100%
“My family would approve of my child getting the HPV vaccine.”		
**Agree**	95.4%	95.4%
“My religious institution would approve of my child getting the HPV vaccine.”		
**Agree**	81.8%	77.3%
**Susceptibility:**		
“My child is likely to get a genital HPV infection in his/her lifetime.”		
**Agree**	45.4%	50%
“My child is likely to develop HPV-related cancer in his/her lifetime.”		
**Agree**	18.2%	22.7%
“My child is likely to develop genital warts in his/her lifetime.”		
**Agree**	18.2%	13.6%

^*^Wilcoxon Signed-Rank tests compared pre-posttest responses for HPV and HPV vaccination attitudes.

**Figure 2:**
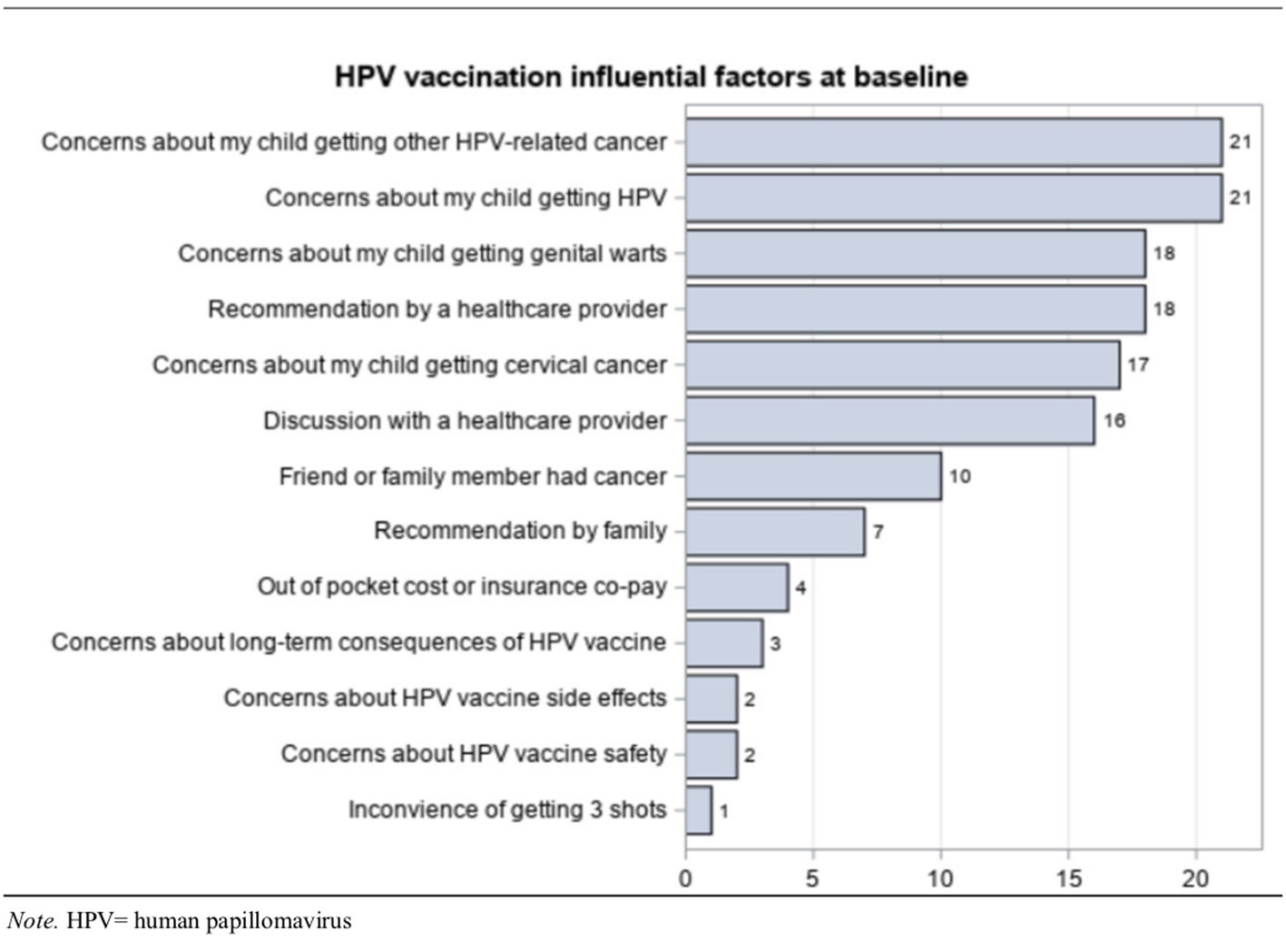
HPV vaccination influence.

At baseline, participants identified factors that influenced their HPV vaccination decision. “Concerns about my child getting other HPV-related cancer” and “concerns about my child getting HPV” were the most frequently identified statements (Figure 2).

#### Participants Described Why They Supported HPV Vaccination

Parents supported the HPV vaccination as cancer prevention. According to one participant, “my daughter was immunized for HPV: Vaccine vs. cervical cancer. The choice is clear.” Another parent said, “I support the HPV vaccine because I'm all for cancer prevention.” Among these supporters of HPV vaccination, the parents agreed, “any opportunity to prevent cancer is foolish not to take advantage of.” Some participants shared a personal connection to HPV or HPV-related cancer that increased their commitment to vaccination. According to one participant, “…I've seen how devastating cervical cancer is. My aunt's MIL got it in her forties. She had so many complications from the cancer and treatments and ended up dying within 2 years of being diagnosed.” Another parent wrote, “…being someone who has HPV, I am definitely for the vaccine. My daughter has already had the vaccine and my son will when he is old enough.” Prior to the intervention, participants already knew that HPV was ubiquitous and that it caused precancerous cervical lesions, as well as cancer. For example, one participant wrote, “I support the vaccine because I think it is important! HPV is so prevalent and yet easy to protect yourself.” Another parent supported the vaccine because, “I've known too many young women scared because of precancerous cells due to HPV.” Among this group of parents, HPV vaccination was common sense. According to one parent, “it seems obvious to me that if you have a means to protect yourself and your children then you should take advantage of it, therefore, vaccinate.”

#### Participants Described Barriers to HPV Vaccination Among Parents

Parents discussed conversations they had in their everyday lives about HPV vaccination. Participants revealed the most common barriers to HPV vaccination among parents in their social networks. Misinformation about HPV vaccination emerged related to safety and side effects, gender, understanding of risk, and sexual activity. A number of participants described vaccine hesitancy toward specific vaccines, including the HPV vaccination. According to one parent:

A lot of people I talk to seem to break vaccines down into two groups, the ones that they consider absolutely necessary (tetanus, polio) and the ones that they consider less important/optional (chicken pox, mumps). Often HPV is grouped in the second category and the reasoning is often based on fear of side effects combined with a lack of appreciation of the impacts of the diseases.

Another participant concurred, “while they are generally pro-vax they've heard that this one has a lot of documented injuries associated with it.” Parents described hearing comments about injuries and deaths related to the HPV vaccine.

Understanding the HPV vaccine as a gender-specific vaccine was also a prominent theme among parents in the participants' social networks. According to one participant, “parents of boys don't see how it applies to their son at all.” Participants also believed that many parents continued to link the vaccine with sexual activity. Parents explained how this opinion impacted vaccine decision-making, “it isn't relevant for preteens since it is sexually transmitted,” and “their kid is too young for sex yet anyway.” According to one participant, “I usually hear it is because their kid is not sexually active or out of fear that will condone their younger child to become sexually active.” Another parent offered a compassionate response to this concern:

I have heard many mothers say they didn't want their child to get the vaccine because they weren't sexually active and didn't plan to become sexually active for years. A pediatric nurse practitioner that I work with has always had the best response to that with “Your daughter is a princess but she may not marry a prince 1 day.” That usually resonates with parents and they end up choosing to vaccinate.

Participants suggested parents who have opted for the vaccine have not been as vocal about it as those with concerns. The prevalence of the virus itself and HPV-related cancers was also overlooked in parents' social networks. According to one participant, “as a parent, I know cancer is a worry for pretty much all parents. But I don't think parents realize how common HPV related cancers are.”

### Intervention Engagement and Treatment Satisfaction

The electronic newsletter showed moderate penetration with an average of 47.2% of participants opening the bi-weekly email (see Table 1). The majority of participants opened emails about “What is HPV?” (68.2%), “Who should get the HPV vaccine?” (54.5%), and “How can I get involved in HPV vaccination efforts in our state?” (63.6%). On the private Facebook page, all posts had one or more interactions by participants (i.e., like, reaction, or comment). On average, there were 3.3 comments per post on the private Facebook page.

Overall, participants rated the intervention positively (see Table 6). All participants (100%) indicated that they enjoyed participating in the intervention. Almost all participants (90.9%) found the bi-weekly emails and posts on Facebook to be valuable, indicated that the Facebook group was useful in helping them learn about HPV vaccination, and reported that they would recommend the program to a friend. Most participants reported that the bi-weekly emails were useful in helping them learn about HPV vaccination (86.4%) and the majority of parents found the webinars to be valuable (68.2%). Most parents (86.4%) found the training valuable in helping them become more confident in starting conversations (online or in-person) about HPV vaccination).

**Table 6:** Intervention experience.

**Items**	**Post** **(*n* = 22)** **%**
How much participants **enjoyed** participating in the program.	100%
**Extremely**	
How **useful** the Facebook group was in helping participants learn about Human Papillomavirus (HPV) vaccination.	90.9%
**Extremely**	
How **helpful** or **valuable** participants found the posts on Facebook.	90.9%
**Extremely**	
How much participants would **recommend** the program to a friend.	90.9%
**Extremely**	
How **helpful** or **valuable** participants found the bi-weekly emails.	90.9%
**Extremely**	
How **useful** the bi-weekly emails were in helping participants learn about HPV vaccination.	86.4%
**Extremely**	
How **helpful** or **valuable** the training was in helping participants to become more confident in starting conversations (online and/or in-person) about HPV vaccination.	86.4%
**Extremely**	
How **helpful** or valuable participants found the Webinars.	68.2%
**Extremely**	

#### Participants Reported High Intervention Engagement and Treatment Satisfaction

Through the online focus groups and open-ended responses on the posttest and longitudinal follow-up surveys, parents unanimously described the benefits of the intervention. Participants appreciated the ease of use and convenience of the private Facebook group, which streamlined seamlessly with their existing social media habits. According to one participant, “I liked the interaction and instruction on Facebook.” Participants demonstrated an increase in knowledge about HPV vaccination. One participant wrote, “I definitely know a lot more about HPV and the HPV vaccine (especially with regard to its impact on men).” Parents described improved confidence and the ability to talk more freely with other parents. According to one participant, “one of the main benefits to me was getting confident with the facts about the HPV vaccine and HPV vaccination rates in S.C.” Another parent wrote, “it has helped to open conversations and given me the opportunity to educate others.” The longitudinal follow-up showed that participants were still using the training 6 months later by filming videos as advocates, joining advocacy groups, and holding many discussions about HPV vaccination with the people in their lives. The training also offered unexpected opportunities for participants to improve communication about HPV vaccination with their children and their patients. According to one participant, “I am a nurse practitioner and going through this program helped me relay to my patients the importance of the HPV vaccine.” Participants expressed gratitude for the program and emphasized how useful it was to them personally. They particularly enjoyed the aspects of the program focused on improving online and in-person communication and suggested the program include more of this practical skills-based training, such as crafting social media posts.

## Discussion

To our knowledge, this is the first evaluation of a collaborative online learning environment to train and support parents to serve as champions for HPV vaccination. The format and content of this technology-mediated intervention was well-accepted by participants. Results indicate the 3-month training program increased knowledge about HPV and HPV vaccination. Overall, there was a statistically significant difference in the average of correct answers from pretest to posttest (*p* = 0.0042) (Table 4). In line with previous studies, participants reported that the intervention addressed important gaps in knowledge about men's susceptibility to HPV and the link between HPV and oropharyngeal and other head and neck cancers (42). At the start of the intervention, the majority of participants were in the action stage of intention to vaccinate their child against HPV. Following the intervention, two participants changed from the contemplation stage to the preparation stage. As anticipated in a sample of participants who were already committed to increasing HPV vaccination prior to the intervention, changes in knowledge, attitudes, and behavioral intentions were modest.

Participants' intervention engagement and treatment satisfaction indicate that this approach provides utility and scalability among parents. All participants (100%) indicated that they enjoyed participating in the intervention. The electronic newsletter showed moderate penetration with an average of 47.2% of participants opening the bi-weekly email. However, this information was repeated in the private Facebook group, offering two ways for participants to engage with the material at their convenience. All Facebook posts had one or more interactions by participants with an average of 3.3 comments per post. Parents reported that Facebook was easy to use, convenient, and provided an optimal platform for instruction and interaction. This finding expands existing research demonstrating that interactive forums empowered parents to express vaccine concerns and offered opportunities to provide answers in real time (16).

Participants described how other parents in their social networks displayed vaccine hesitancy toward the HPV vaccination despite an overall pro-vaccination attitude. This finding builds on limited research aimed at determining different types of vaccine hesitancy (43). Parents reported misinformation about HPV vaccination related to safety and side effects, gender, understanding of risk, and sexual activity that remained prevalent in their social networks. Results reflect recent research that safety concerns were the most common reason parents chose not to start the HPV vaccine for unvaccinated adolescents (44). In line with our formative audience research, this study also identified misinformation related to gender, understanding of risk, and sexual activity as barriers to HPV vaccination (31, 32). Results provide evidence that health communicators and public health professionals should consider using social media platforms to disseminate science-based information about the safety of vaccines (7). In the current study, the majority of participants reported using multiple devices every day (59.1%) with 22.7% reporting hourly use of multiple devices, indicating that parents are reachable online.

Most parents (86.4%) believed that the intervention resulted in improved confidence and the ability to talk more freely about HPV vaccination with other parents in-person and on social media. This finding supports the effectiveness of connecting vaccine-interested parents with those who are like-minded in order to assist them in countering vaccine hesitancy and misinformation on social media (6). This study offers an innovative approach to effectively address the spread of rumors about HPV vaccination on social media (43). Participants suggested parents in their social networks who have opted for the vaccine have not been as vocal about it as those with concerns. This research answers the call to action by researchers to assist parents who support vaccination to speak out easily and often by providing information that is simple and shareable online (6).

### Study Limitations and Strengths

This mixed methods study offers an innovative approach to reach parents to overcome barriers to HPV vaccination. Participants represented counties across four regions of South Carolina. However, the homogeneity of the population and small sample size limit the generalizability of the results. Future studies should purposively sample diverse parents, especially in terms of race and ethnicity. Researchers may consider less stringent screening criteria, increased participant incentives, more targeted recruitment, and a budget for recruitment to improve diversity in future studies. Another limitation of the study is the largely single gender population of mothers. Although future studies may seek to incorporate more fathers, it is important to continue to target women since mothers serve as the primary decision makers for adolescents receiving HPV vaccination (13–15). It is also important to focus on raising the voices of women who support vaccination because the majority of participants on anti-vaccination Facebook pages are women (11).

This feasibility study offers a model of cultivating HPV vaccination champions in a community setting and demonstrates potential for scalability and dissemination of this intervention approach (24–27). In the context of the pandemic, this technology-mediated intervention offers an innovative model to combat the proliferation of anti-science and anti-vaccine messaging. Specifically, the automated delivery of bi-weekly emails and Facebook posts offers an opportunity to scale the intervention among larger groups of parents with limited resources. Participants demonstrated high treatment satisfaction and robust engagement in one of the first technology-mediated HPV vaccination training programs for parents. This study benefited from a high retention rate and longitudinal evaluation, including a 6-month follow-up survey. The use of objective measures and qualitative assessment of the intervention, including online focus groups, were additional study strengths.

## Conclusion

A technology-mediated intervention for parents increased their confidence and motivated them to speak more freely about HPV vaccination in-person and online with others in their social networks. The collaborative online learning environment cultivated HPV vaccination champions through a 3-month training program that supported parents to serve as proponents and social media influencers to overcome barriers to HPV vaccination. Participants identified prevalent misinformation about HPV vaccination and learned how to effectively craft messages to address concerns related to safety and side effects, gender, understanding of risk, and sexual activity. Objective measures and qualitative open-ended assessment showed high intervention engagement and treatment satisfaction. The effectiveness of this feasibility study suggests that social media is an appropriate platform to reach parents with HPV vaccination information that is simple and shareable in-person and online. This study combined education and health promotion messages with skills-based communication training to empower parents to raise their voices in support of HPV vaccination.

## Data Availability Statement

The datasets presented in this article are not readily available because the informed consent procedure guaranteed participants in this study that only authorized study staff would have access to the data. Requests to access the datasets should be directed to BLS@cofc.edu.

## Ethics Statement

The studies involving human participants were reviewed and approved by The Institutional Review Board (IRB) at the College of Charleston, Charleston, SC. The patients/participants provided their written informed consent to participate in this study.

## Author Contributions

BS and KC contributed to conception and design of the study. NR organized the database and assisted with project management. AW assisted with project management and conducted quantitative data analysis. BS performed the qualitative analysis. BS and NR wrote the first draft of the manuscript. All authors contributed to manuscript revision, read, and approved the submitted version.

## Conflict of Interest

The authors declare that the research was conducted in the absence of any commercial or financial relationships that could be construed as a potential conflict of interest.
